# Enamel Matrix Derivative Promote Primary Human Pulp Cell Differentiation and Mineralization

**DOI:** 10.3390/ijms15057731

**Published:** 2014-05-05

**Authors:** Elisabeth Aurstad Riksen, Maria A. Landin, Sjur Reppe, Yukio Nakamura, Ståle Petter Lyngstadaas, Janne E. Reseland

**Affiliations:** 1 Department of Biomaterials, Institute for Clinical Dentistry, University of Oslo, Blindern, N-0317 Oslo, Norway; E-Mails: elisaba@odont.uio.no (E.A.R.); maria.landin@odont.uio.no (M.A.L.); yukiomalmo@hotmail.com (Y.N.); spl@odont.uio.no (S.P.L.); 2 Department of Medical Biochemistry, Oslo University Hospital, N-0450 Oslo, Norway; E-Mail: sjur.reppe@medisin.uio.no

**Keywords:** pulp cells, enamel matrix derivative (EMD), bone, dentin, epigenetic factors

## Abstract

Enamel matrix derivative (EMD) has been found to induce reactive dentin formation; however the molecular mechanisms involved are unclear. The effect of EMD (5–50 μg/mL) on primary human pulp cells were compared to untreated cells and cells incubated with 10^−8^ M dexamethasone (DEX) for 1, 2, 3, 7, and 14 days in culture. Expression analysis using Affymetrix microchips demonstrated that 10 μg/mL EMD regulated several hundred genes and stimulated the gene expression of proteins involved in mesenchymal proliferation and differentiation. Both EMD and DEX enhanced the expression of amelogenin (amel), and the dentinogenic markers dentin sialophosphoprotein (DSSP) and dentin matrix acidic phosphoprotein 1 (DMP1), as well as the osteogenic markers osteocalcin (OC, BGLAP) and collagen type 1 (COL1A1). Whereas, only EMD had effect on alkaline phosphatase (ALP) mRNA expression, the stimulatory effect were verified by enhanced secretion of OC and COL1A from EMD treated cells, and increased ALP activity in cell culture medium after EMD treatment. Increased levels of interleukin-6 (IL-6), interleukin-8 (IL-8), and monocyte chemoattractant proteins (MCP-1) in the cell culture medium were also found. Consequently, the suggested effect of EMD is to promote differentiation of pulp cells and increases the potential for pulpal mineralization to favor reactive dentine formation.

## 1. Introduction

Dental pulp cells have the potential to differentiate into odontoblasts that forms dentin both during odontogenesis and reparative/reactive dentin after completion of root formation. [1]. Deposition of protective reactive dentin by the induction of hard-tissue deposition is essential to maintain pulpal health and prevent hypersensitivity or necrosis after injury or conservative therapy. This effect is currently obtained by applications of calcium hydroxide in endodontic and dental traumatology [2].

Epithelium derived tooth enamel proteins induce mesenchymal cell differentiation and commercial preparations of porcine fetal enamel matrix derivative (EMD) is used clinically for regeneration of periodontal defects, which includes new cementum formation, new alveolar bone formation, and new periodontal ligament regeneration [3,4]. EMD is reported to stimulate a process mimicking normal odontogenesis where epithelial derived and mesenchymal tissue reciprocally interact [5], and can thereby serve as a biologically active pulp-dressing agent that specifically induces pulpal wound healing and hard-tissue formation without negatively affecting the healthy pulp [6,7]. During dentin formation, odontoblasts synthesize both collagen and several non-collagenous proteins like dentin sialophosphoprotein (DSSP) and secrete these into the dentin extracellular matrix with subsequent matrix mineralization and crystal growth [8]. Previous studies have shown that both pulpal stem cells from late and early developmental stages are able to differentiate toward cells of the osteo-/odontoblastic lineage, induce alkaline-phosphatase-activity and produce calcified matrix, however this ability was gradually lost during culturing [9].

Dexamethasone (DEX) is found to induce formation of odontoblast-like cells from cultured human dental pulp cells on dentin *in vitro* and is currently an established method used to stimulate differentiation to odontoblast-like phenotype in pulp cell cultures [10].

A recent study on human adult dental pulp showed that a stem cell niche with differentiation potential might exist in the dental pulp primary cell culture, and that their phenotypes may be altered towards osteoblast- or odontoblast-like cells. The dentinogenic markers had peak levels of expression around passage 5 and were limited to early passages before 8–9, whereas osteoblastic markers were found in all passages [11].

Clinical trials have shown that partially pulpotomized EMD gel-treated teeth had significantly less tooth hypersensitivity compared to Ca(OH)_2_-treated [12] with a positive effect on wound healing/new tissue and hard tissue formation [13].

The mechanism by which EMD influences odontoblastic/osteoblastic differentiation is not well understood. A previous report suggests that EMD may directly stimulate odontoblasts or pulp cells to produce collagen matrix for calcification [14]. It was also hypothesized that the presence of transforming growth factor (TGF) -β1 or amelogenin peptides in EMD may induce cell signaling that stimulates matrix formation and mineralization [15]. Bone morphogenetic protein 2 and 4 (BMP 2/4) have been reported to contribute to the induction of biomineralization and this effect were reduced by noggin, an antagonist of BMP [16], and BMP-expressing macrophages induced by EMD might play important roles in reparative dentin formation [17]. Recent studies suggest that combination of capping materials with EMD would increase the quality of capping by increasing biocompatibility of capping materials like Ca(OH)_2_ to induce pulpal healing parallel with calcification [18]. A previous *in vivo* study has shown that the enamel protein ameloblastin significantly increased intrapulpal calcification compared to Ca(OH)_2_ and was suggested to be the biologically active agent in EMD-induced reparative dentin formation [19]. Dentin mineralization occurs when calcium is transported from the circulation by transmembraneous transport in the odontoblasts alongside non-collagenous macromolecules [20]. Dentin sialophosphoprotein (*DSSP*) is important in biomineralization and expression has been demonstrated in both hard and soft tissues, like bone and the pericytes of blood vessels in the dental pulp [21]. Other non-collagenous macromolecules important for mineralization of dentin and bone are dentin matrix proteins (DMP1s) and recent studies indicate that different forms of DMP1 may work collectively in controlling the mineralization process regarding nucleation and crystal growth [22].

The aim of the present study was to identify possible molecular mechanisms related to EMD’s effect on dentin formation and wound healing using primary human pulp cells and odontoblasts *in vitro*. Affymetrix microchip analysis and Real Time quantitative Reverse Transcription PCR (qRT-PCR) were performed to analyze changes in gene regulation by EMD whereas changes in protein secretion from human pulp cells and odontoblasts was identified using Luminex.

## 2. Results

### 2.1. Affymetrix Microarray Expression Analysis

EMD treatment had effect on more than 533 genes measured by microarray analysis. Several major cellular pathways were calculated by up- and down-regulation of mRNA expression relative to untreated control and exponential effects of gene expressions as log ratio in Table 1.

Positive effects were found on molecules regulating anti-apoptosis concomitant with down-regulation of molecules that stimulates apoptosis and phagocytosis indicating decreased programmed cell death (PCD) and atrophy.

Ankylosis inorganic pyrophosphate transport regulator (*ANKH*) regulating transport and tissue calcification was up-regulated (*p* = 0.008).

Up-regulation of genes involved in cell adhesion; contactin associated protein-like 5 (*CNTNAP5*) (*p =* 0.0002) and neurofascin (*NFASC*) (*p =* 0.0005) were found, as well as effect on cell cycle like GTP binding by stimulation of septin 1 (*SEPT1*) (*p =* 0.003). Effect on chemokine activity was confirmed by up-regulation of interleukin 8 (IL-8) (*p =* 0.0002) and IL-11 (*p =* 0.0002).

Growth factors; bone morphogenetic protein 4 (*BMP4*) (*p =* 0.027), osteoglycin (*OGN*) (*p =* 0.095), platelet derived growth factor D (*PDGFD*) (*p =* 0.0002) and fibroblast growth factor 7 (*FGF7*) (*p =* 0.0002) were down-regulated, whereas transcription factor binding to immunoglobulin heavy constant mu (IGHM) enhancer 3 (*TFE3*) (*p =* 0.03) was enhanced by EMD treatment. TFE3 is involved in the TGF beta signaling pathway and promotes TGF beta effects and aberrant TFE3 transcription activity is involved in the pathogenesis of alveolar soft-part sarcoma (ASPS) [23].

Transcription factors important to neural development and post-traumatic healing were up-regulated including General transcription factor IIIA (*GTF3*) (*p =* 0.046) involved in neurogenesis [24] and Forkhead box protein G1 (*FOXG1B*) (*p =* 0.038) linked to development of hippocampal dentate gyrus and CNS structures [25]. This is relevant as the dental pulp is a sensory organ.

Factors stimulating microtubule cytoskeleton including microtubule-based movement, protein transport, vesicle-mediated transport, cell mitosis, and proliferation were up-regulated by EMD treatment (Table 1).

Topmost regulated molecules after Affymetrix expression analysis were identified by Ingenuity Pathway Analysis (IPA) (Ingenuity Systems) as high mobility group AT-hook 2 (*HMGA2*) (*p =* 0.0015), C-type lectin domain family 16, member A (*CLEC16A*) (*p =* 0.001), transmembrane protease, serine 7 (*TMPRSS7*) (*p =* 0.0009), grainyhead-like 1 (*GRHL1*) (*p =* 0.0007), slingshot protein phosphatase 2 (*SSH2*) (*p =* 0.0005), contactin associated protein-like 5 (*CNTNAP5*) (*p =* 0.0002), chromosome 10 open reading frame 140 (*C10ORF140*) (*p =* 0.002), Fanconi anemia, complementation group B (*FANCB*) (*p =* 0.000005), neurofascin (*NFASC*) (*p =* 0.0005), dedicator of cytokinesis 5 (*DOCK5*) (*p =* 0.0005) (Table 1).

### 2.2. Gene Expression Analyzed by Real-Time PCR

**Figure 1.**
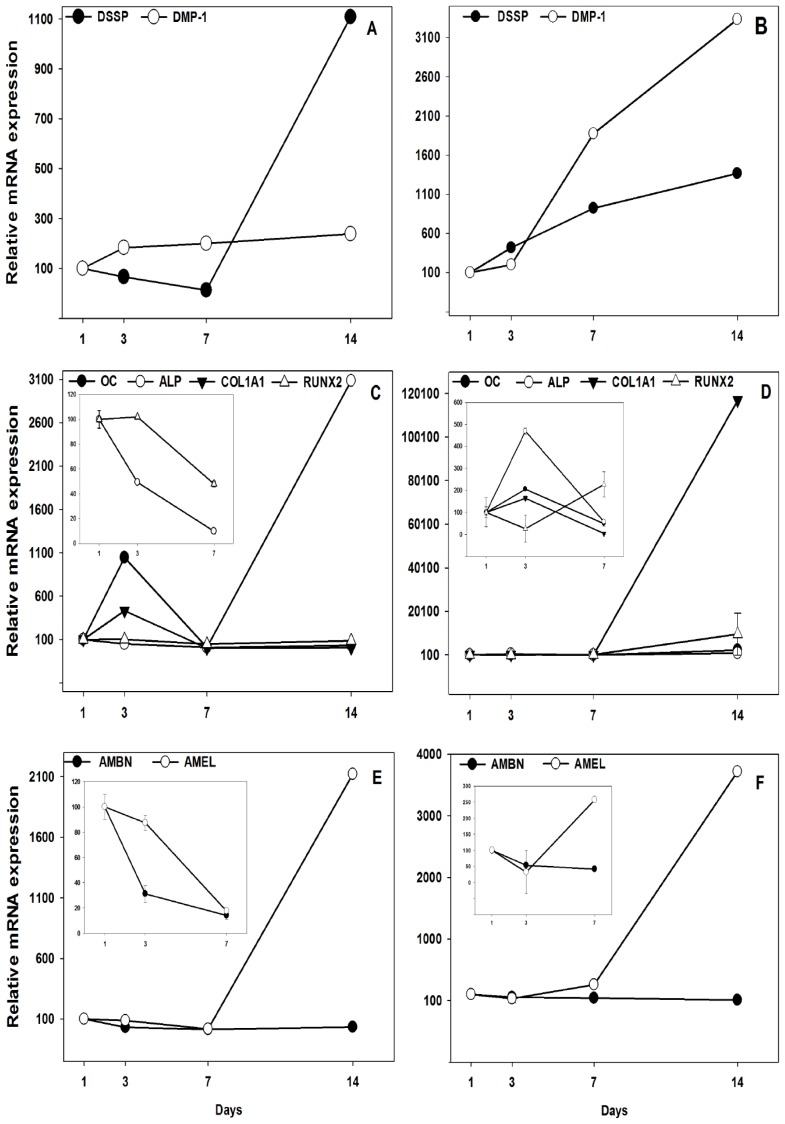
Effect of Enamel matrix derivative (EMD) (10 μg/mL) on mRNA expression of dentin sialophosphoprotein (DSSP) and dentin matrix acidic phosphoprotein 1 (DMP-1) (**A**) (left panel) and dexamethasone (DEX) (10^−8^ M) (**B**) (right panel), osteocalcin (OC), alkaline phosphatase (ALP), runt-related transcription factor 2 (RUNX2) and collagen type 1 (COL1A1) (**C**) (left panel) and DEX (10^−8^ M) (**D**) (right panel); and amelogenin (AMEL) and ameloblastin (AMBN) (**E**) (left panel) and DEX (10^−8^ M) (**F**) (right panel) in primary human pulp cells. The data is presented as mean ± SD and calculated in percentage of untreated cells (100%) at each time point.

In cells treated with EMD (10 μg/mL) the gene expression of *DSPP* initially decreased after three and seven days of incubation (*p* = 0.002 and *p* ≤ 0.001, respectively) followed by a significant (*p* ≤ 0.001) 11-fold increase at day 14. *DMP-1* expression was upregulated by 2-fold increase at all time points after 10 μg/mL EMD treatment (*p* ≤ 0.001, *p* ≤ 0.001, and *p* ≤ 0.001, respectively) (Figure 1A, left panel). An increase in odontoblast specific genes and differentiation towards odontoblasts, were confirmed by a similar increase in *DSPP* and *DMP-1* expression in cells treated with DEX (Figure 1B, right panel).

EMD (10 μg/mL) induced reduced expression of alkaline phosphatase (*ALP*) at day three and seven (*p* ≤ 0.001 and *p* ≤ 0.001, respectively), and significant 31-fold enhancement (*p* ≤ 0.001) at day 14 (Figure 1C, left panel) compared to untreated control. After three days incubation with 10 μg/mL EMD the *OC* gene expression was 11-fold higher (*p* ≤ 0.001), and *COL1A1* expression was 5-fold higher (*p* ≤ 0.001) compared to the expression in untreated pulp cells. Both *OC* and *COL1A1* expression were reduced after EMD treatment for 7 and 14 days (*p* ≤ 0.001 and *p* ≤ 0.001, respectively) (Figure 1C, left panel). In cells treated with DEX both *OC* (*p* ≤ 0.001) and *COL1A1* (*p* ≤ 0.001) expression was doubled after three days incubation, whereas after 14 days of DEX treatment *OC* expression was enhanced 22-fold (*p* ≤ 0.001) and *COL1A1* 1200-fold (*p* ≤ 0.001) (Figure 1D, right panel) compared to untreated cells. No effect on runt-related transcription factor 2 (*RUNX2*) expression was found after three days, but *RUNX2* were reduced after 7 and 14 days (*p* ≤ 0.001 and *p* = 0.003, respectively) treatment with EMD (Figure 1D, right panel). DEX doubled the *RUNX2* expression after seven days (*p* ≤ 0.001) and induced a 96-fold increase after 14 days (*p* ≤ 0.001) of incubation (Figure 1D, right panel).

EMD reduced the expression of *AMEL* at day 3 and 7 (*p* = 0.047 and *p* ≤ 0.001, respectively) and enhanced the expression more than 21-fold (*p* ≤ 0.001) at day 14 compared to control, whereas the *AMBN* expression were reduced (*p* ≤ 0.001 for all) at all time points tested (Figure 1E, left panel). The equivalent effects were found in pulp cells after DEX treatment (Figure 1F, right panel) confirming that EMD induced an expression profile of enamel proteins similar to an odontoblastic phenotype.

### 2.3. Proteins in Culture Medium Measured by Elisa and Luminex

**Figure 2.**
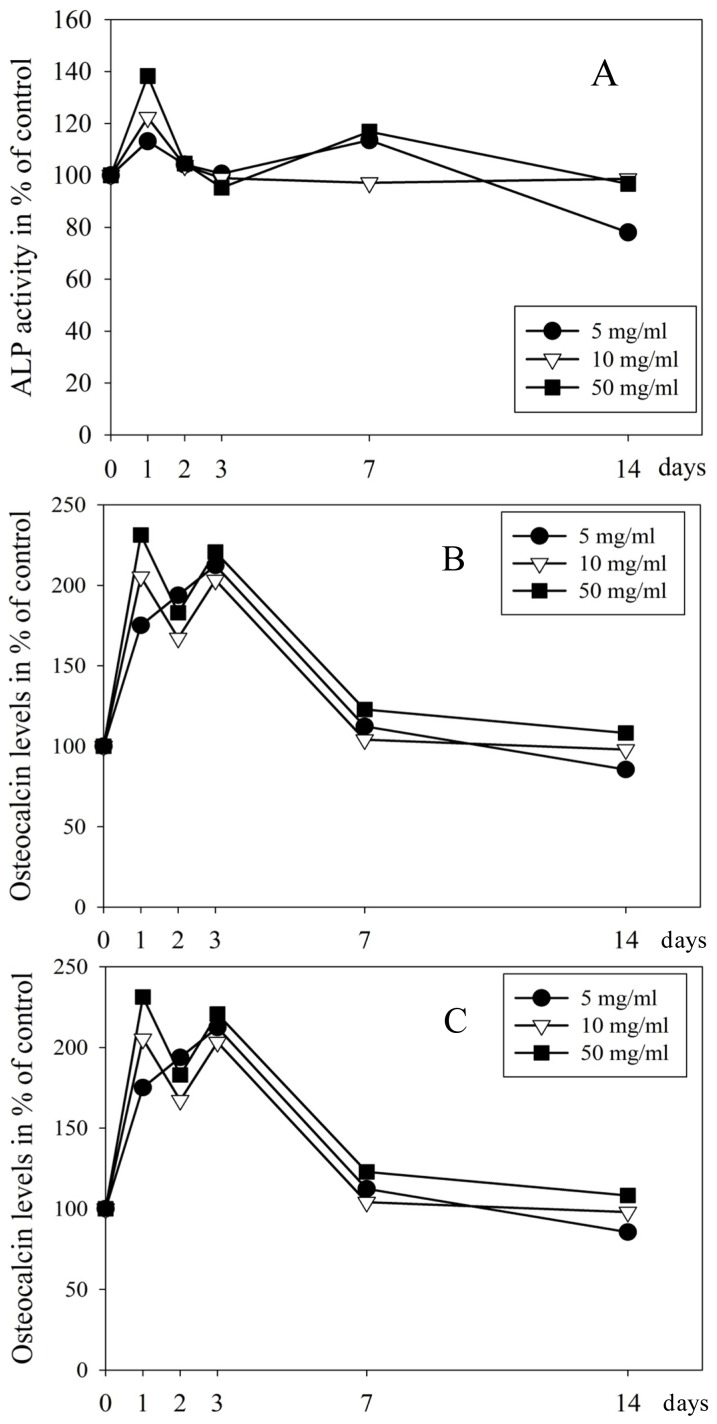
Effect of EMD (5, 10, 50 μg/mL) on the alkaline phosphatase (ALP) activity (**A**); osteocalcin (OC) (**B**); and collagen type 1 (COL1A1) secretion (**C**) in the cell medium by primary human pulp cells. The data is presented as mean ± SD in percent of untreated cells (100%) at each timepoint.

After one-day incubation 50 μg/mL EMD induced a 1.4-fold increase in the ALP activity (ALP/TP) (*p =* 0.04) in the cell culture medium (Figure 2A). No significant effects were observed at other time points by any of the EMD concentrations tested (Figure 2A).

EMD treatments (5, 10, and 50 μg/mL) induced an up to 2.5-fold increase in OC secretion (*p* = 0.017, *p* < 0.001, and *p* < 0.001, respectively) at day one, a 1.8-fold at day two (*p =* 0.009, *p* ≤ 0.001, and *p* ≤ 0.001, respectively) and a 2.2-fold at day three (*p* ≤ 0.001, *p* ≤ 0.001, and *p* ≤ 0.001, respectively) compared to control (Figure 2B).

COL1A levels in the medium were enhanced by EMD (5, 10, 50 μg/mL); up to 3.5-fold at day one (*p* ≤ 0.001, *p =* 0.010, and *p* ≤ 0.001, respectively), and 2.5-fold at day two (*p =* 0.030, *p* ≤ 0.001, and *p =* 0.009, respectively) compared to control. No significant changes were found at day three (Figure 2C). No effect of EMD was found on OC and collagen type 1 secretion from pulp cells after 7 and 14 days of incubation (data not shown).

**Figure 3.**
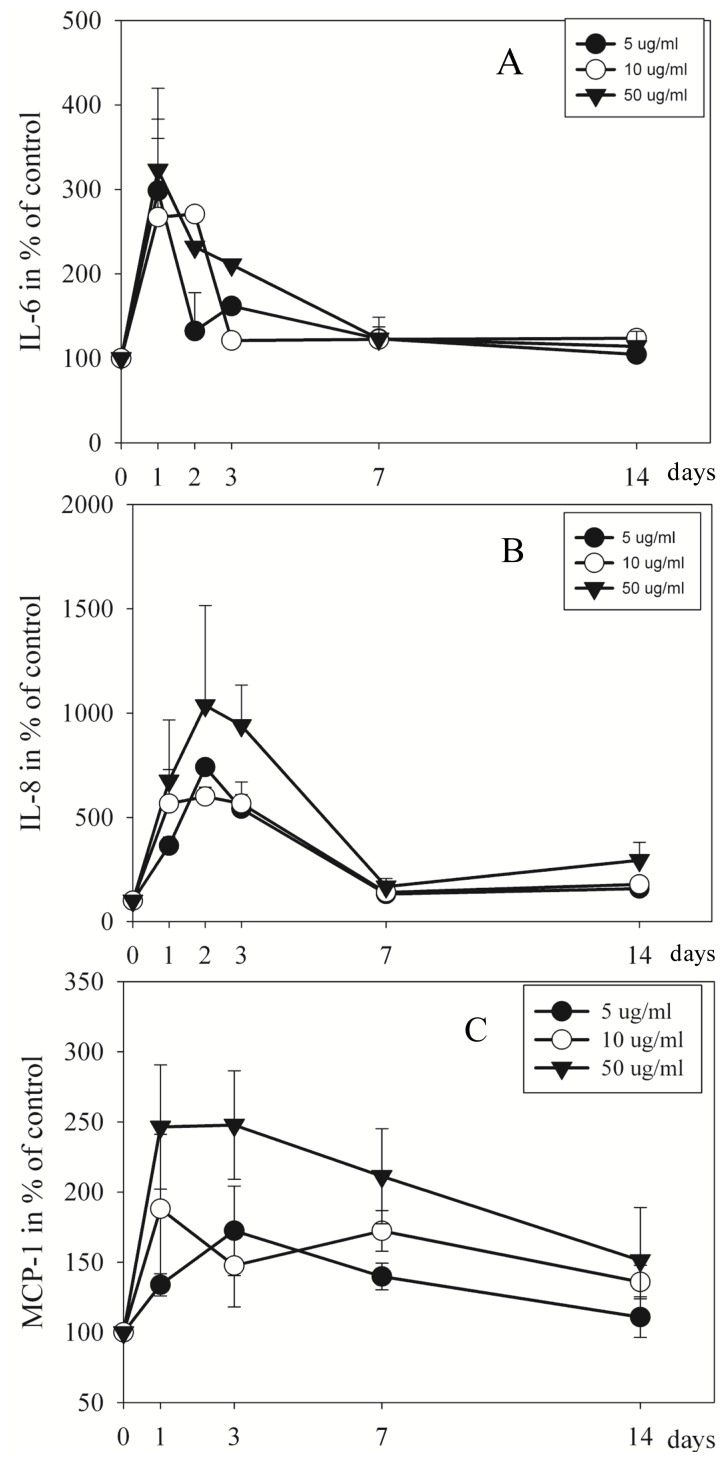
The effect of 5, 10, 50 μg/mL of EMD on the concentration of IL-6 (**A**); IL-8 (**B**); and MCP-1 (**C**) in the cell culture medium. The data are presented as mean ± SD and calculated in percent of untreated cells (100%) at each time point.

EMD (5, 10, 50 μg/mL) induced a 3-fold increase in IL-6 secretion to the medium after one day (*p =* 0.042, *p =* 0.037, and *p =* 0.041, respectively) and two days (*p* ≤ 0.001, and *p* ≤ 0.001, respectively) (Figure 3A).

Whereas IL-8 secretion was increased 7-fold after one day (10 and 50 μg/mL EMD; *p =* 0.023 and *p =* 0.013, respectively), 10-fold after two days (5, 10, and 50 μg/mL; *p =* 0.028, *p =* 0.034, and *p =* 0.006, respectively), 9-fold after three days (*p =* 0.002, *p =* 0.003, and *p* ≤ 0.001, respectively) compared to control (Figure 3B).

The secretion of MCP-1 was enhanced 3-fold after one day (10 and 50 μg/mL; *p =* 0.035 and *p =* 0.004, respectively), 2-fold after two days (5, 10, and 50 μg/mL; *p =* 0.022, *p =* 0.014, and *p =* 0.002, respectively), 3-fold after three days (5 and 50 μg/mL; *p =* 0.009 and *p =* 0.001, respectively) (Figure 3C).

### 2.4. No Cytotoxic Effects Was Discovered

There were no changes in the Lactate dehydrogenase (LDH) activity in the cell culture medium after EMD treatment of the primary pulp cells (data not shown) at any of the time points tested, indicating that EMD at the concentrations used here had no major cytotoxic effects on the primary human pulp cells.

## 3. Discussion

### 3.1. Affymetrix Analysis Showed Effect on Transcription Factors and DNA Repair

Affymetrix microarray expression analysis has previously shown that EMD activate the expression of a high number of genes in the primary human osteoblasts [26], and we found the similar pattern in pulp cells. RUNX2, a transcriptional factor regulating, e.g., OC, was not altered, whereas v-maf avian musculoaponeurotic fibrosarcoma oncogene homolog (MAF) was down-regulated. MAF and RUNX2 are cooperating in differentiation of osteoblasts in favor of adipocytes however this differentiation switch is still unclear [27]. Expression of FANCB, a gene encoding proteins involved in protection and repair of DNA damage [28], was increased.

### 3.2. EMD Had Positive Effect on Genes Involved in both Neural and Mesenchymal Healing

*HMGA2* were highly expressed in pulp cells 24 h after EMD treatment. This is a gene that is a transcriptional regulating factor that induces differentiation into certain mesenchymal cell lineages [29] and is suggested to have stimulating effect on bone mineral density (BMD) and osteogenesis [30].

An *in vitro* study on gene profile in periodontal ligament cells (PDL) demonstrated positive effect of EMD on genes involved in protein synthesis and mineralization ability [31]. This confirms similar effect of EMD on PDL and pulp cells, both tissues that mainly consists of fibroblasts and cells capable of mineralization.

*ANKH* regulates intracellular and extracellular levels of pyrophosphate/inorganic phosphate critical for maintaining mineral homeostasis and dysfunction of *ANKH* gene is linked to Craniometaphysial dysplasia (CMD) [32]. Increased ANKH expression indicates that EMD increases the levels of pyrophosphate/inorganic phosphate, thus, facilitating mineralization.

Evolutionary it is significant that pulp is a part of the sensory dental organ. During odontogenesis reciprocal stimulation and effect between ecto-mesenchymal tissue and neural tissue is crucial for development of the dental pulp. After pulp injury regulation of innervation and inflammation is important to preserve pulpal health trough both regulation of re-innervation and intrapulpal tissue pressure. Significant markers for neural development up-regulated in pulp cells after EMD treatment were CLEC16A [33] with effect on activation and control of neuroinflammation, CNTNAP5 involved in CNS development [34] and NFASC that regulate neural development and axon innervations [35].

The Affymetrix analysis indicates that EMD have positive effects on genes that regulates hard tissue mineralization and facilitates innervations of the pulp. These findings support clinical trials finding that EMD induces less postoperative pain and tooth hypersensitivity [12] and increases reparative/tertiary dentin formation and intrapulpal mineralization [13].

### 3.3. EMD Induces Both Expression and Secretion of Osteogenic Proteins and Increased Mineralization Potential

Both qRT-PCR and Enzyme-linked immunosorbent assay (ELISA) demonstrated positive effect on pulp cells with enhanced gene up-regulation and secretion of OC and COL1A and increased ALP activity after EMD treatment. EMD has previously been shown to have similar stimulatory effect on osteoblasts [26]. EMD had a dose dependent effect on both expression and secretion of osteogenic markers, such as OC and COL1A, and mineralization potential trough increased ALP activity in pulp cells; indicating a dose specific effect on osteogenic proteins and increased mineralization in the pulp tissue.

Gene array of primary human osteoblasts exposed to EMD have in a previous study demonstrated that EMD up-regulates several differentiation markers including collagen isoforms among COL1A and ALP as well as growth factors like VEGF [36]. Our study indicates a similar effect of EMD on gene expression in osteoblasts and in pulp cells.

Human pulp cells were stimulated by EMD to up-regulate expression of DSSP and DMP-1 similar to DEX treatment; an acknowledged method to stimulate transformation of pulpal pluripotential stem cells to differentiate to odontoblasts [10]. Primary dental pulp contains approximately two percent stem cells able to differentiate to mineralizing cells [37]. In pulp tissue these genes are up- and down-regulated during growth and healing leading to both innate dentinogenic potential and osteogenic potential. RUNX2 expression was found to be unchanged following EMD treatment. This is supported by a previous study that demonstrated no effect of EMD on the mRNA levels of RUNX2, but changed the location of expression from the cytoplasm to the nucleus in osteoblasts [38]. The pulp cells exhibit differentiation trough expression of genes DSSP, DMP-1, ALP and OC markers for mineralizing tissue like bone and dentin [11]. OC and DSSP have previously been found in both odontoblasts and mineralizing dentin [39], and DMP-1 has been found to regulate the promoter for both DSPP and OC [40]. Our study demonstrated effect on gene expression level on DSPP, DMP-1, ALP, and OC after EMD treatment. Thus our findings indicate that EMD has potent positive effect on differentiation of pulp cells capable of inducing hard tissue formation. EMD as the product Emdogain^®^ has clinically been used to enhance bone healing after destructive marginal periodontitis [41] by inducing up-regulation of genes involved mesenchymal proliferation and differentiation favoring bone formation over bone resorption [42]. We speculate that EMD has potential for other applications like pulp capping or sealing before restorative treatment by stimulating pulpal mineralization and less hypersensitivity and postoperative pain.

### 3.4. EMD Has Immunoregulatory Effect on Pulp Cells

EMD had an acute stimulatory effect on the expression (IL-8, IL-11) and secretion (IL-6, IL-8 and MCP-1) of cytokines that mediates both inflammatory and vascular responses. IL-6 has immunomodulating effect and might be both a pro- and anti inflammatory cytokine. Anti-inflammatory effect is achieved by regulation of IL-1β and TNF-α, the major inflammatory cytokines, and previous studies have found that IL-6 suppress IL-1 (α, β) mRNA in CNS [43] and is essential for TNF-α responses like fever [44]. In our study, we found that neither IL-1β nor TNF-α were elevated after EMD treatment. No effect on other immunostimulatory cytokines like IL-10, INF-γ and GM-CSF were found indicating no pro-inflammatory effect of EMD and is supported by a cDNA microarray study on PDL cells that concluded that EMD down-regulates the expression of genes involved in the early inflammatory phases of wound healing while simultaneously up-regulating genes encoding growth and repair-promoting molecules [45].

Previous studies have demonstrated that DMP-1 (10 ng/mL) that is involved in intrapulpal mineralization stimulates the production of IL-6 and IL-8 from pulp fibroblasts [46], and we might speculate that increased DMP-1 expression might trigger increased IL-6 and IL-8 secretion. Other studies have shown that IL-6 and IL-8 expression have immunomodulatory properties of mesenchymal stem cells in periapical lesions [47]. *In vivo* studies have shown that IL-11 together with IL-6 is required for normal bone remodeling [48] and IL-11 supposed to have osteotrophic properties and mediates anti-inflammatory responses to major injury [49]. A recent study has shown that IL-11 inhibits adipogenic differentiation and stimulates osteoblastogenic differentiation from human bone marrow mesenchymal stem cells and increases ALP activity [50].

MCP-1 was increased after EMD treatment in pulp cells indicating effect on vascularization. MCP-1 acts as a chemokine to recruit mononuclear cells to the dental follicle and an important vascular signaling factor [51].

EMD treatment in concentrations used has stimulatory effect on pulp cells *in vitro* inducing gene expression and secretion of factors promoting tissue healing and mineralization.

## 4. Experimental Section

### 4.1. Cell Culture and Treatment

Commercially available primary human pulp cells (passage 5) from unerupted intact human third molars (DPK-DTPC-H, Dominion Pharmakine, Derio, Spain) were grown in DMEM medium (PAA laboratories, Pashing, Austria) containing 10% fetal bovine serum, 50 U/mL penicillin and 50 μg/mL streptomycin. The cultures were incubated at 37 °C in a humidified atmosphere of 95% air and 5% CO_2_. The medium was changed three times weekly and the cells were sub-cultured and seeded in 6-well plates in duplicates. EMD was dissolved in 10% acetic acid to a stock solution of 10 mg/mL prior to dilution in cell culture medium to final concentration. The cells were incubated with 5, 10, or 50 μg/mL EMD (Batch 9121, Biora, Malm? Sweden) was obtained directly from manufacturer Biora as freeze-dried protein in tubes. EMD was dissolved directly in cold cell culture medium before it was incubated at 37 °C prior to administration.

In parallel, cells treated with dexamethasone (DEX) (10^−8^ M) to regulate the commitment to form odontoblast-like cells [52], were used as positive control, whereas untreated cells were used as negative control. Cells and cell culture medium were harvested after 1, 2, 3, 7, and 14 days. All cell culture treatments were performed three individual times.

### 4.2. Purification of RNA, Microarray Analysis by Affymetrix

The effect of EMD on gene expression in primary human pulp cells was studied after 24 h incubation with 10 μg/mL EMD and performed three individual times. RNA was isolated using TRIZOL reagent (Life Technologies, Gaithersburg, MD, USA) according to manufacturer instructions and further purified using the RNeasy kit (Qiagen Inc., Valencia, CA, USA) in order to get rid of organic components.

Double stranded cDNA and biotin labeled cRNA probes were made from 5 μg total RNA using the Superscript Choice system (Invitrogen life technologies, Carlsbad, CA, USA) and the Bioarray (Enzo Biochem, New York, NY, USA), respectively. Procedures were according to recommendations from Affymetrix (Santa Clara, CA, USA). cRNA was hybridized to HG-U133A microchips (Affymetrix) containing cDNA oligonucleotides representing more than 22,000 transcripts, followed by washing and staining on the Gene Chips Fluidics Station 450 (Affymetrix) according to manufacturer instructions. The chips were scanned on the Affymetrix Gene Array 2500 scanner. The quality of the RNA and probe was controlled by an Affymetrix based test measuring the ratio between 5′ and 3′ mRNAs for β-actin and glyceraldehyde-3-phosphate dehydrogenase (GAPDH) and found to be satisfactory. The datasets were processed by the Affymetrix Mas5.0 software, and signal values representing the expression level of each transcript were generated.

TATA binding protein (TBP) mRNA was included in the reactions and used as internal standard. TBP was verified not to be differentially expressed before and after treatment with parathyroid hormone (PTH) from Affymetrix expression analysis. Predesigned primers and a probe labeled with the reporter fluorescent dye VIC, specific for TBP, were used. For the Real time rtPCR, cDNA was amplified under the following conditions: 50 °C for 2 min, and 94.5 °C for 10 min, followed by 40 cycles at 97 °C for 30 s and 59.7 °C for 1 min. The relative mRNA amount of each gene was calculated using the Comparative CT Method “Separate Tubes” following the instructions; PE Applied Biosystems and adjusted for the expression of TBP mRNA.

### 4.3. Alkaline Phosphatase (ALP) Activity

ALP activity was quantified by measuring the hydrolysis of *para*-Nitrophenylphosphate (pNPP) (Sigma Diagnostics, St. Louis, MI, USA) at 405 nm. Standard curves, constructed using calf intestinal alkaline phosphatase (CIAP) (Promega, Madison, WI, USA), were run in parallel for quantification purposes and performed three individual times. The total protein content in the culture medium was determined using Sigma Microprotein PR assay kit with a Protein Standard Solution Calibrator (Sigma Diagnostics). Analyses were performed using a Cobas Mira chemistry analyzer (Roche Diagnostics, Basel, Switzerland). Intra-assay and inter-assay variability were less than 2.4% and 3.2%, respectively. The assay detection range was 10–2000 mg/L. ALP activity was calculated in terms of nmol of pNPP/min/mg of total protein in each individual sample. The activity in cell culture medium was finally expressed in % of the negative controls at each of the time points.

### 4.4. mRNA Isolation Prior to Real Time RT-PCR Quantification

Cells were lysed in lysis/binding buffer (100 mM Tris-HCl, pH 8.0, 500 mM LiCl, 10 mM EDTA, pH 8.0, 0.5 mM dithiothreitol (DTT), and 1% sodium dodecyl sulfate (SDS)). mRNA was isolated using magnetic beads (oligo (dT)_25_) as described by the manufacturer (Dynal AS, Oslo, Norway). Beads containing mRNA were resuspended in 10 mM Tris-HCl, pH 8.0, and stored at −70 °C until use. 1 μg of mRNA was used to synthesize first-strand complementary DNA (cDNA) using RevertAid™ First Strand cDNA Synthesis kit (Fermentas, St. Leon Route, Germany).

### 4.5. Real-Time PCR Quantification of Target Genes for Dental Biomineralization

Real-time RT-PCR was performed in a CFX384 (Bio-Rad, Hercules, CA, USA) using the SsoAdvanced SYBR Green Green Supermix (Bio-Rad). The thermal profile was as follows: denaturing at 98 °C for 5 min followed by 40 cycles with denaturing at 95 °C for 10 s annealing 60 °C for 60 s, primer extension at 72 °C for 30 s. Reactions were performed in triplicates on a 384-well plate. The primer sequences for the target genes (runt-related transcription factor 2 (*RUNX2*), osteocalcin (*OC*), *COL1A1*, *ALP*, dentin sialophosphoprotein (*DSPP*), dentin matrix acidic phosphoprotein 1 (*DMP1*), amelogenin (*AMEL*), ameloblastin (*AMBN*)) are listed in Table 2. The housekeeping gene glyceraldehyde-3-phosphate dehydrogenase (GAPDH) was used as reference. The efficiency of each set of primers was between 95% and 100%. The real time RT-PCR data was analyzed using the Δ*C*_t_ method, with efficiency (E) correction for the individual transcripts. Where relative expression = (E_target_)^Δ^*^C^*^t^_target(control-sample)_/(E_ref_)^Δ^*^C^*^t^_ref(control-sample)_.

### 4.6. Analysis of the Cell Culture Medium

Enzyme-linked immunosorbent assay (ELISA) was used to quantify the release of osteocalcin (OC) (BioSource, Camarillo CA, USA) and procollagen I type A (COL1A) (Takara Bio Inc., Ohtsu-shi, Japan) to the cell culture medium collected from primary human pulp cell cultures according to manufacturer’s description. Intra-assay and inter-assay variability were less than 2.9% and 3.5%, respectively. The assay detection range was 0–65 ng/mL (OC) and 0–640 ng/mL (COL1A). OC and COL1A concentrations were calculated relative to the amount of total protein in each sample and presented as % untreated cells at the different time points.

### 4.7. Cytokine Levels in the Culture Medium

Multianalyte profiling was performed using the Luminex-100 system and the XY Platform (Luminex Corporation, Austin, TX, USA). Calibration microspheres for classification and reporter readings as well as sheath fluid were also purchased from Luminex Corporation. The acquired fluorescence data were analyzed by the STarStation software (Version 2.0; Applied Cytometry Systems, Sheffield, UK). Prior to analysis, the samples were concentrated 10 times using Microsep™ Centrifugal tubes with 3 k Dada cut-off from Pall Life Science (Ann Armor, MI, USA). The concentrations of different cytokines; granulocyte-macrophage colony-stimulating factor (GM-CSF), interferon (IFN-α, IFN-γ), interleukins (IL-1β, IL-Ra, IL-2, IL-2R, IL-4, IL-5, IL-6, IL-7, IL-8, IL-10, IL-12p40/p70, IL-13, IL-15, IL-17), interferon gamma-induced protein (IP-10), monocyte chemotactic protein-1 (MCP-1), monokine induced by gamma interferon (MIG), macrophage inflammatory proteins (MIP-1α, MIP-1β), tumor necrosis factor-alpha (TNF-α), Eotaxin-1 (CCL11), and RANTES (CCL5) in the cell culture medium were determined using the Human Cytokine 20-plex from BioSource (Carmarillo, CA, USA). All analyses were performed according to the manufacturers’ protocols.

### 4.8. Lactate Dehydrogenase (LDH) Activity

Cell viability was confirmed by monitoring the release of lactate dehydrogenase (LDH) into the medium. LDH was measured using the microplate based Cytotoxicity Detection Kit (LDH) (Boehringer, Mannheim, Germany). According to the manufacturer’s protocol, 50 μL aliquots of cell culture medium were used and absorbance read on a microplate reader (Asys expert 96, Asys Hitech, Eugendorf, Austria) at 450 nm. Analysis were performed on three individual cellular experiments.

### 4.9. Statistics

Statistical evaluation of real-time RT-PCR data was carried out using graphPad Instat (Graphpad Sofware Inc., La Jolla, CA, USA), whereas SigmaStat software (Systat Software Inc., San Jose, CA, USA) were used for the protein analysis. Protein analysis data passed normality and equal variance tests. Statistical comparison between groups and treatments was performed using parametric test one-way analysis of variance (one-way ANOVA) and *post hoc* Holm-Sidak tests. A probability of less than or equal to 0.05 was considered significant. Data were presented as a percentage of untreated cells (=100%) at each time point of observation.

## 5. Conclusions

EMD have stimulating effects on expression of genes involved in mesenchymal proliferation and differentiation in pulp cells measured by Affymetrix microarrays and qRT PCR. This promotive effect on factors involved in differentiation, signalling and mineralisation were confirmed on protein level by ELISA, Luminex and ALP/TP. This *in vitro* study indicate some of the molecular mechanisms involved in EMD’s stimulatory effect on wound healing in pulp tissue. This is an *in vitro* study to describe potential effect in the dental pulp and adjacent dentin with the limitations of the study design, thus, further studies must be done *in vivo* to confirm effect on dental organ level.

## Figures and Tables

**Table 1.** Topmost regulated molecules after Affymetrix expression analysis (log ratio).

Biological Response	Probe Set Identification (Affymetrix)	Signal Log Ratio	Gene Symbol	Gene Title
Apoptosis	204614_at	1, 8	SERPINB2	serine (or cysteine) proteinase inhibitor, clade B (ovalbumin)
	204467_sat	−1	SNCA	synuclein, alpha (non A4 component of amyloid precursor)
	205681_at	1, 5	BCL2A1	BCL2-related protein A1
	202095_s_at	1, 4	BIRC5	baculoviral IAP repeat-containing 5 (survivin)
	204237_at	−1	GULP1	GULP, engulfment adaptor PTB domain containing 1

Cell adhesion	205679_x_at	−1, 2	AGC1	aggrecan 1 (chondroitin sulfate proteoglycan 1)
	1553418_aat	3, 8	CNTNAP5	contactin associated protein-like 5
	222853_at	−1, 1	FLRT3	fibronectin leucine rich transmembrane protein 3
	243645_at	3, 5	NFASC	neurofascin
	205908_s_at	−1, 5	OMD	osteomodulin
	204649_at	1, 2	TROAP	trophinin associated protein (tastin)
	238871_at	−1, 7	MLLT4	Myeloid/lymphoid or mixed-lineage leukemia
	242472_x_at	−3, 7	FNBP4	Formin binding protein 4
	231726_at	−1	PCDHB14	protocadherin beta 14
	214111_at	1, 1	OPCML	opioid binding protein/cell adhesion molecule-like

Cell cycle	227552_at	1, 8	sep.01	septin 1
	204456_s_at	−1	GAS1	growth arrest-specific 1
	204991_s_at	1	NF2	neurofibromin 2 (bilateral acoustic neuroma)
	211094_s_at	−1, 8	NF1	neurofibromin 1 (neurofibromatosis)
	225123_at	−1, 1	SESN3	Sestrin 3
	202859_x_at	1, 9	IL8	interleukin 8

Growth factor	222722_at	−1, 4	OGN	osteoglycin (osteoinductive factor, mimecan)
	219304_s_at	−1, 5	PDGFD	platelet derived growth factor D
	205782_at	−1, 1	FGF7	fibroblast growth factor 7 (keratinocyte growth factor)
	211518_s_at	−1, 3	BMP4	bone morphogenetic protein 4

Transcription	1569098_sat	−1, 2	TP53BP1	tumor protein p53 binding protein, 1
	203359_s_at	1, 1	MYCBP	c-myc binding protein
	201417_at	−1	SOX4	SRY (sex determining region Y)-box 4
	227162_at	−1, 1	ZBTB26	zinc finger and BTB domain containing 26
	1568873_at	1, 2	ZNF519	zinc finger protein 519
	221530_s_at	−1, 2	BHLHB3	basic helix-loop-helix domain containing, class B, 3
	204915_s_at	1	SOX11	SRY (sex determining region Y)-box 11
	206018_at	1, 4	FOXG1B	forkhead box G1B

microtubule cytoskeleton	202890_at	−1	MAP7	microtubule-associated protein 7
	206364_at	1	KIF14	kinesin family member 14
	221258_sat	1, 4	KIF18A	kinesin family member 18A
	219570_at	−1	C20orf23	chromosome 20 open reading frame 23
	219306_at	1, 1	KIF15	kinesin family member 15
	209408_at	1, 2	KIF2C	kinesin family member 2C
	218755_at	1, 1	KIF20A	kinesin family member 20A
	210052_sat	1, 2	TPX2	TPX2, microtubule-associated protein homolog (Xenopus laevis)
	219588_sat	1, 1	MTB	more than blood homolog
	204162_at	1, 2	KNTC2	kinetochore associated 2
	209680_sat	3, 1	KIFC1	kinesin family member C1
	38158_at	1, 4	ESPL1	extra spindle poles like 1 (S. cerevisiae)
	218009_sat	1, 3	PRC1	protein regulator of cytokinesis 1
	2047at	1, 4	KIF23	kinesin family member 23
	225540_at	−1, 8	MAP2	Microtubule-associated protein 2

**Table 2.** Sequences of real-time PCR (RT-PCR) primers.

Gene	Sequence of Left Primer	Sequence of Right Primer
AMELX	5′-CTCATCACCACATCCCAGTG-3′	5′-TGTTGGATTGGAGTCATGGA-3′
AMBN	5′-AGAGCACAGTGCATGTCAGC-3′	5′-AAGAACGGCACTGCAAAACT-3′
DSPP	5′-GGGAAAGTGGTGGTGGTGCT-3′	5′-CACCAGGGCATGGCTGTAAG-3′
DMP1	5′-AAGCAGACAGCGAATCCAGT-3′	5′-CTGCTGAGCTGCTGTGAGAC-3′
OC	5′-GCAAGTAGCGCCAATCTAGG-3′	5′-GCTTCACCCTCGAAATGGTA-3′
ALP	5′-GACAAGAAGCCCTTCACTGC-3′	5′-AGACTGCGCCTGGTAGTTGT-3′
COL1A1	5′-CATCTCCCCTTCGTTTTTGA-3′	5′-CCAAATCCGATGTTTCTGCT-3′
RUNX2	5′-TTACTTACACCCCGCCAGTC-3′	5′-CACTCTGGCTTTGGGAAGAG-3′
GAPDH	5′-CTCTGCTCCTCCTGTTCGAC-3′	5′-ACGACCAAATCCGTTGACTC-3′

## References

[1] Stanley H.R., Pereira J.C., Spiegel E., Broom C., Schultz M. (1983). The detection and prevalence of reactive and physiologic sclerotic dentin, reparative dentin and dead tracts beneath various types of dental lesions according to tooth surface and age. J. Oral Pathol.

[2] Mohammadi Z., Dummer P.M. (2011). Properties and applications of calcium hydroxide in endodontics and dental traumatology. Int. Endod. J.

[3] Hammarstrom L. (1997). Enamel matrix, cementum development and regeneration. J. Clin. Periodontol.

[4] Heijl L., Heden G., Svardstrom G., Ostgren A. (1997). Enamel matrix derivative (emdogain) in the treatment of intrabony periodontal defects. J. Clin. Periodontol.

[5] Gestrelius S., Lyngstadaas S.P., Hammarstrom L. (2000). Emdogain-periodontal regeneration based on biomimicry. Clin. Oral Investig.

[6] Nakamura Y., Hammarstrom L., Matsumoto K., Lyngstadaas S.P. (2002). The induction of reparative dentine by enamel proteins. Int. Endod. J.

[7] Nakamura Y., Slaby I., Matsumoto K., Ritchie H.H., Lyngstadaas S.P. (2004). Immunohistochemical characterization of rapid dentin formation induced by enamel matrix derivative. Calcif. Tissue Int.

[8] Butler W.T. (1995). Dentin matrix proteins and dentinogenesis. Connect. Tissue Res.

[9] Takeda T., Tezuka Y., Horiuchi M., Hosono K., Iida K., Hatakeyama D., Miyaki S., Kunisada T., Shibata T., Tezuka K. (2008). Characterization of dental pulp stem cells of human tooth germs. J. Dent. Res.

[10] Huang G.T., Shagramanova K., Chan S.W. (2006). Formation of odontoblast-like cells from cultured human dental pulp cells on dentin *in vitro*. J. Endod..

[11] Min J.H., Ko S.Y., Cho Y.B., Ryu C.J., Jang Y.J. (2011). Dentinogenic potential of human adult dental pulp cells during the extended primary culture. Hum. Cell.

[12] Kiatwateeratana T., Kintarak S., Piwat S., Chankanka O., Kamaolmatyakul S., Thearmontree A. (2009). Partial pulpotomy on caries-free teeth using enamel matrix derivative or calcium hydroxide: A randomized controlled trial. Int. Endod. J.

[13] Olsson H., Davies J.R., Holst K.E., Schroder U., Petersson K. (2005). Dental pulp capping: Effect of emdogain gel on experimentally exposed human pulps. Int. Endod. J.

[14] Ishizaki N.T., Matsumoto K., Kimura Y., Wang X., Yamashita A. (2003). Histopathological study of dental pulp tissue capped with enamel matrix derivative. J. Endod.

[15] Kawase T., Okuda K., Momose M., Kato Y., Yoshie H., Burns D.M. (2001). Enamel matrix derivative (EMDOGAIN) rapidly stimulates phosphorylation of the MAP kinase family and nuclear accumulation of smad2 in both oral epithelial and fibroblastic human cells. J. Periodontal Res.

[16] Iwata T., Morotome Y., Tanabe T., Fukae M., Ishikawa I., Oida S. (2002). Noggin blocks osteoinductive activity of porcine enamel extracts. J. Dent. Res.

[17] Kaida H., Hamachi T., Anan H., Maeda K. (2008). Wound healing process of injured pulp tissues with emdogain gel. J. Endod.

[18] Guven E.P., Yalvac M.E., Sahin F., Yazici M.M., Rizvanov A.A., Bayirli G. (2011). Effect of dental materials calcium hydroxide-containing cement, mineral trioxide aggregate, and enamel matrix derivative on proliferation and differentiation of human tooth germ stem cells. J. Endod.

[19] Nakamura Y., Slaby I., Spahr A., Pezeshki G., Matsumoto K., Lyngstadaas S.P. (2006). Ameloblastin fusion protein enhances pulpal healing and dentin formation in porcine teeth. Calcif. Tissue Int.

[20] Linde A. (1995). Dentin mineralization and the role of odontoblasts in calcium transport. Connect. Tissue Res.

[21] Prasad M., Butler W.T., Qin C. (2010). Dentin sialophosphoprotein in biomineralization. Connect. Tissue Res.

[22] Gericke A., Qin C., Sun Y., Redfern R., Redfern D., Fujimoto Y., Taleb H., Butler W.T., Boskey A.L. (2010). Different forms of DMP1 play distinct roles in mineralization. J. Dent. Res.

[23] Stockwin L.H., Vistica D.T., Kenney S., Schrump D.S., Butcher D.O., Raffeld M., Shoemaker R.H. (2009). Gene expression profiling of alveolar soft-part sarcoma (ASPS). BMC Cancer.

[24] Wang Y., Lin L., Lai H., Parada L.F., Lei L. (2013). Transcription factor Sox11 is essential for both embryonic and adult neurogenesis. Dev. Dyn.

[25] Tian C., Gong Y., Yang Y., Shen W., Wang K., Liu J., Xu B., Zhao J., Zhao C. (2012). Foxg1 has an essential role in postnatal development of the dentate gyrus. J. Neurosci.

[26] Reseland J.E., Reppe S., Larsen A.M., Berner H.S., Reinholt F.P., Gautvik K.M., Slaby I., Lyngstadaas S.P. (2006). The effect of enamel matrix derivative on gene expression in osteoblasts. Eur. J. Oral Sci.

[27] Nishikawa K., Nakashima T., Takeda S., Isogai M., Hamada M., Kimura A., Kodama T., Yamaguchi A., Owen M.J., Takahashi S. (2010). Maf promotes osteoblast differentiation in mice by mediating the age-related switch in mesenchymal cell differentiation. J. Clin. Investig.

[28] Meier D., Schindler D. (2011). Fanconi anemia core complex gene promoters harbor conserved transcription regulatory elements. PLoS One.

[29] Morshedi A., Ren Z., Li J., Droge P. (2013). Probing into the biological processes influenced by ESC factor and oncoprotein HMGA2 using iPSCS. Stem Cell Rev.

[30] Kuipers A., Zhang Y., Cauley J.A., Nestlerode C.S., Chu Y., Bunker C.H., Patrick A.L., Wheeler V.W., Hoffman A.R., Orwoll E.S. (2009). Association of a high mobility group gene (HMGA2) variant with bone mineral density. Bone.

[31] Barkana I., Alexopoulou E., Ziv S., Jacob-Hirsch J., Amariglio N., Pitaru S., Vardimon A.D., Nemcovsky C.E. (2007). Gene profile in periodontal ligament cells and clones with enamel matrix proteins derivative. J. Clin. Periodontol.

[32] Zhang H., Somerman M.J., Berg J., Cunningham M.L., Williams B. (2007). Dental anomalies in a child with craniometaphysial dysplasia. Pediatr. Dent.

[33] Wu X., Li J., Chen C., Yan Y., Jiang S., Shao B., Xu J., Kang L., Huang Y., Zhu L. (2012). Involvement of CLEC16A in activation of astrocytes after LPS treated. Neurochem. Res.

[34] Djurovic S., Gustafsson O., Mattingsdal M., Athanasiu L., Bjella T., Tesli M., Agartz I., Lorentzen S., Melle I., Morken G. (2010). A genome-wide association study of bipolar disorder in norwegian individuals, followed by replication in icelandic sample. J. Affect Disord.

[35] Kriebel M., Wuchter J., Trinks S., Volkmer H. (2012). Neurofascin: A switch between neuronal plasticity and stability. Int. J. Biochem. Cell. Biol.

[36] Miron R.J., Bosshardt D.D., Zhang Y., Buser D., Sculean A. (2013). Gene array of primary human osteoblasts exposed to enamel matrix derivative in combination with a natural bone mineral. Clin. Oral Investig.

[37] Coppe C., Zhang Y., Den Besten P.K. (2009). Characterization of primary dental pulp cells *in vitro*. Pediatr. Dent..

[38] Miron R.J., Oates C.J., Molenberg A., Dard M., Hamilton D.W. (2010). The effect of enamel matrix proteins on the spreading, proliferation and differentiation of osteoblasts cultured on titanium surfaces. Biomaterials.

[39] Bronckers A.L., Farach-Carson M.C., van Waveren E., Butler W.T. (1994). Immunolocalization of osteopontin, osteocalcin, and dentin sialoprotein during dental root formation and early cementogenesis in the rat. J. Bone Miner. Res.

[40] Narayanan K., Gajjeraman S., Ramachandran A., Hao J., George A. (2006). Dentin matrix protein 1 regulates dentin sialophosphoprotein gene transcription during early odontoblast differentiation. J. Biol. Chem.

[41] Lyngstadaas S.P., Wohlfahrt J.C., Brookes S.J., Paine M.L., Snead M.L., Reseland J.E. (2009). Enamel matrix proteins; Old molecules for new applications. Orthod. Craniofac. Res.

[42] Yan X.Z., Rathe F., Gilissen C., van der Zande M., Veltman J., Junker R., Yang F., Jansen J.A., Walboomers X.F. (2012). The effect of enamel matrix derivative (emdogain^®^) on gene expression profiles of human primary alveolar bone cells. J. Tissue Eng. Regen. Med.

[43] Chai Z., Alheim K., Lundkvist J., Gatti S., Bartfai T. (1996). Subchronic glucocorticoid pretreatment reversibly attenuates IL-beta induced fever in rats; IL-6 mRNA is elevated while IL-1 alpha and IL-1 beta mRNAs are suppressed, in the CNS. Cytokine.

[44] Sundgren-Andersson A.K., Ostlund P., Bartfai T. (1998). IL-6 is essential in TNF-alpha-induced fever. Am. J. Physiol.

[45] Parkar M.H., Tonetti M. (2004). Gene expression profiles of periodontal ligament cells treated with enamel matrix proteins *in vitro*: Analysis using cdna arrays. J. Periodontol.

[46] Abd-Elmeguid A., Yu D.C., Kline L.W., Moqbel R., Vliagoftis H. (2012). Dentin matrix protein 1 activates dental pulp fibroblasts. J. Endod.

[47] Dokic J., Tomic S., Cerovic S., Todorovic V., Rudolf R., Colic M. (2012). Characterization and immunosuppressive properties of mesenchymal stem cells from periapical lesions. J. Clin. Periodontol.

[48] Sims N.A., Jenkins B.J., Nakamura A., Quinn J.M., Li R., Gillespie M.T., Ernst M., Robb L., Martin T.J. (2005). Interleukin-11 receptor signaling is required for normal bone remodeling. J. Bone Miner. Res.

[49] Schinkel C., Wick M., Muhr G., Koller M. (2005). Analysis of systemic interleukin-11 after major trauma. Shock.

[50] Nawa K., Ikeno H., Matsuhashi N., Ogasawara T., Otsuka E. (2013). Discovering small molecules that inhibit adipogenesis and promote osteoblastogenesis: Unique screening and Oncostatin M-like activity. Differentiation.

[51] Gu L., Tseng S.C., Rollins B.J. (1999). Monocyte chemoattractant protein-1. Chem. Immunol.

[52] Alliot-Licht B., Bluteau G., Magne D., Lopez-Cazaux S., Lieubeau B., Daculsi G., Guicheux J. (2005). Dexamethasone stimulates differentiation of odontoblast-like cells in human dental pulp cultures. Cell Tissue Res.

